# Shear Wave and Strain Elastography in Crohn’s Disease—A Systematic Review

**DOI:** 10.3390/diagnostics11091609

**Published:** 2021-09-03

**Authors:** Anna Grażyńska, Jakub Kufel, Arkadiusz Dudek, Maciej Cebula

**Affiliations:** 1 Józef Dietl Specialist Hospital in Cracow, Skarbowa 4, 31-121 Cracow, Poland; grazynska.anna@gmail.com (A.G.); a.dudekan@gmail.com (A.D.); 2 Department of Biophysics, Faculty of Medical Sciences in Zabrze, Medical University of Silesia in Katowice, Jordana 18, 40-043 Zabrze, Poland; jakubkufel92@gmail.com; 3 Department of Radiodiagnostics, Invasive Radiology and Nuclear Medicine, Department of Radiology and Nuclear Medicine, School of Medicine in Katowice, Medical University of Silesia, Medyków 14, 40-752 Katowice, Poland

**Keywords:** Crohn’s disease, sonoelastography, shear wave elastography, strain elastography

## Abstract

One modern imaging technique used in the diagnosis of Crohn’s disease (CD) is sonoelastrography of the intestine. Guidelines regarding the use of bowel sonoelastography in CD have still not been specified. The aim of our research was to conduct a systematic review of the use of sonoelastography in the diagnosis, assessment, and monitoring of strictures in the course of CD. A systematic review was conducted according to the PRISMA guidelines statement. The following databases were searched in January 2021: MEDINE via PubMed, Embase and Scopus. The search utilised the following MeSH tags: ‘Ultrasound Shear Wave’, ‘Elastography’, ‘elastogram’, ‘elastographies’ AND ‘Crohn disease’. The inclusion criteria were as follows: from 2010 or later, articles with abstracts, articles in English, human-based studies and original articles. Articles were assessed independently by two reviewers. Out of 181 articles, only 15 met the criteria and were included in the review. Due to a small number of studies and significant methodological differences, the feasibility of using sonoelastography for Crohn’s disease must be proven through further research and analysis. In the future, standardised assessment criteria and cut-off points should be established for both strain elastography (SE) and shear wave elastography (SWE).

## 1. Introduction

Crohn’s disease (CD) is a form of transmural granulomatous inflammation that can occur in any portion of the digestive tract. However, the most frequently observed location of Crohn’s disease is in the intestines. Lesions develop in an intermittent pattern of affected and unaffected segments, resulting in fibrosis, destruction of the bowel wall, strictures, obstructions, abscesses and fistulas. Inflammation spreads centrifugally, starting in the mucosa and gradually spreading toward external layers, eventually affecting the entire bowel wall [1,2,3]. Despite state-of-the-art modern pharmacotherapy involving biological drugs, complications from CD still may necessitate extensive surgical treatment [4,5,6]. Endoscopy is the well-established diagnostic standard for inflammatory bowel diseases. Simultaneously, aside from many advantages, it has also several limitations. This method is an invasive, time-consuming technique, and its results depend on proper patient preparation [7,8,9,10]. Moreover, the proximal intestine is difficult to assess. Inflammatory strictures are not always visible during an endoscopy. Another example of a limit in this technique is the detection of fistulas. They are frequently omitted, and biopsies do not tend to reach sufficiently deep into the bowel wall [11,12,13]. Although all imaging diagnostic methods can find inflammatory processes based on the increased after-contrast vascularisation, detecting bowel fibrosis remains a diagnostic challenge. Magnetic resonance enterography (MRE) and computer tomographic enterography (CTE) are moderately accurate and require a high degree of enteral contrast, diminishing patient comfort.

Moreover, the technical limitations of MRE and additional exposure of the patient to high-dose radiation in CTE must be considered [14,15,16]. Another diagnostic technique in CD is contrast-enhanced ultrasound (CEUS), which is based on administering intravenous contrast during a standard ultrasonographic (US) assessment. CEUS has been applied to the evaluation of CD activity and the differentiation between oedematous and fibrotic strictures. Furthermore, CEUS has also been proven to be feasible in monitoring responses to pharmacotherapy in CD [17].

In addition to the methods described above, ultrasound elastography is a promising, relatively new, non-invasive technique. It utilises ultrasound to assess tissue stiffness. There are two major types of elastography: shear wave elastography (SWE) and real-time elastography (RTE), also known as strain elastography (SE). SWE is based on acoustic radiation force impulse (ARFI), which propagates through tissue and subsequently assesses its elastic properties by measuring the velocity of the shear wave. This method should enable a repeatable, objective and quantitative evaluation of tissue stiffness [18,19]. Diversely, the strain elastography assessment is a derivative of comparison between targeted and surrounding tissues after external pressure induced by an operator. The results from SE are presented as a colour-coded elastogram, which is a map illustrating elastic strains with colour gradation [20,21].

Aside from the differences due to different elastography techniques, there is an emerging issue regarding a lack of standardisation of measurement between the ultrasound systems, rendering the comparison of results impossible. In strain elastography or strain rate imaging, the form of applied force is mechanically induced as the active external displacement of tissue surface or passive internal physiologically induced. Shear wave elastography, on the other hand, can be classified into point shear wave elastography (pSWE) and two-dimensional shear wave elastography (2D-SWE), which is subdivided based on the applied force and imaging method [22].

Point shear wave elastography is offered by manufacturers such as Siemens, Esaote, GE, Hitachi, Philips and Samsung. This method does not result in an elastogram; only a regional average of shear wave speed (SWS) is calculated. The quality of measurement is evaluated via estimation algorithms, which differ between the systems [23].

For 2D-SWE manufacturers such as Siemens, Toshiba, Philips and Mindray, radiation force impulses should be focused at various depths, resulting in a single image within a colour box with or without running refresh. GE offers a method based on a radiation force stimulus in a ‘comb push’ with directional filtering, with a result presented as a single image within a colour box. The Hologic Supersonic uses a radiation force focus sweep over depth faster than shear wave speed to create a Mach cone, resulting in up to several frames per second images within a colour box. Stiffness is measured by putting the region of interest (ROI) in a tissue of interest, where the recommended ROI size differs between manufacturers, with some of them not having an established minimum or maximum ROI size. Due to the above differences in methodology, although results are presented in kPa after the recalculation that is based on the Young modulus, they cannot be compared between the systems [22,23].

At this point, the authors would like to underline that the above summarisation may not be up to date, as the manufacturers are constantly introducing new technological solutions related to elastography measurement. The fast implementation pace raises questions about the validation and reliability of the offered methods.

There are currently no widely approved guidelines for utilising sonoelastography in CD diagnosis. Since the introduction of sonoelastography to clinical use, over a dozen empirical studies have been conducted to evaluate the efficacy and usability of elastography in CD. The results of these findings were partially included in guidelines published by the European Federation of Societies for Ultrasound in Medicine and Biology in 2013 [24]. The other guidelines including the findings were those developed by The European Federation of Societies for Ultrasound in Medicine and Biology Guidelines and Recommendations for the Clinical Practice of Elastography in Non-Hepatic Applications [25]. The authors of the guidelines recommend the application of sonoelastography for the characterisation of intestinal lesions in CD. Moreover, they indicate that SE is the only method which can differentiate between inflammatory and fibrotic strictures. However, elastography has not been included in the latest guidelines published by the American College of Gastroenterology [26] and the European Society for Paediatric Gastroenterology Hepatology and Nutrition [27] related to the diagnosis and treatment of CD.

This article aims to systematically review the use of sonoelastography in the diagnosis, assessment and strictures monitoring of patients suffering from Crohn’s disease. It also aims to identify the obstacles that hinder its clinical adoption; furthermore, we provide insight into what is needed to ensure progress in this field to overcome some of them.

## 2. Materials and Methods

The systematic review was conducted according to the PRISMA guidelines statement [28]. File S1 constitutes an appendix to this article and is a checklist that proves the reliability of the review. The study was not registered.

### 2.1. Search Strategy and Selection Criteria

**Figure 1:**
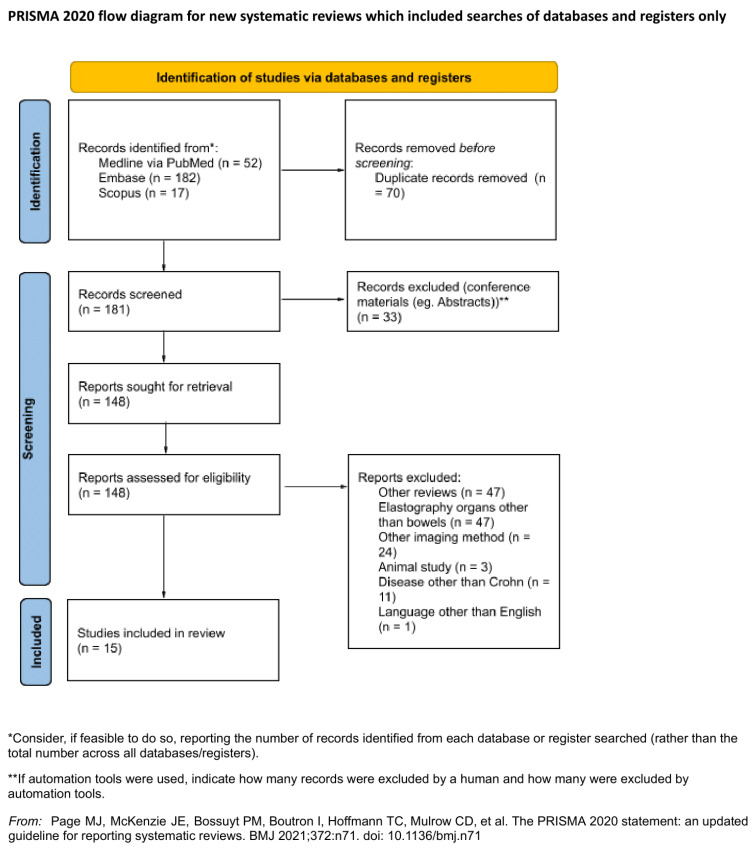
PRISMA flow diagram.

The following databases were searched in January 2021: MEDINE via PubMed, Embase and Scopus. The search was conducted utilising the following MeSH tags: ‘Ultrasound Shear Wave’, ‘Elastography’, ‘elastogram’, ‘elastographies’ AND ‘Crohn’s disease’. The authors reported the search strategy in File S2. The search was filtered using criteria that included the following: from 2010 or later, articles with abstracts, articles in English, human-based studies and original articles. Both included and excluded articles are presented in the PRISMA workflow (Figure 1).

### 2.2. Data Extraction and Quality Assessment

All articles found in medical databases (181) were exported and subsequently imported to Rayyan QCRI (Qatar Computing Research Institute). Articles were assessed independently by two reviewers to prevent mutual selection bias. The utility of the articles for the purposes of the current study was also evaluated. This evaluation assessed the article’s relation to the review topic, information about both CD and sonoelastography, and data for further analysis. Duplicates (71), other systematic reviews (47) and conference papers including abstracts (33) were excluded from the study. Another 86 articles did not meet the inclusion criteria. All contradictory opinions of the two reviewers after unblinding were settled by a third independent researcher. Finally, 15 articles were included in the review. The Kappa Cohen factor was estimated at 0.38 (agreement in 84.43%), which is interpreted as a fair agreement between reviewers [29]. The study and elastography methodology were evaluated; subgroups based on applied sonoelastographic technology, examination protocol, study aim and outcomes were formed for further analysis. No assumption was made for missing or unclear information, and no studies that appeared to meet the inclusion criteria were excluded.

## 3. Results

Out of the 15 articles (Table 1) included in the analysis, 13 are prospective studies [30,31,32,33,34,35,36,37,38,39,40,41,42], one consists of both prospective and retrospective groups [43], and one is a series of clinical case studies [44]. Articles were published between 2011 and 2019. The total number of patients who participated in the studies described in the articles is 507: 427 in prospective studies, 77 in retrospective studies and three in clinical case studies. The average age of the participants was 38.27 (3–90 years) in one article, this data was not supplemented. The age of patients suffering from CD varied between 6 years [36] and 90 years [34]. The average period of illness was 9.05 years. However, five studies did not provide this variable [34,36,41,43,44]. Segments assessed in the studies were as follows: upper part of the digestive tract [37], ileum [33,34,35,36,37,38,40,41,42,43,44], ileum terminal [30,31,39,40,44], ileocolon [31,33,34,35,36,39,41], colon [31,36,38,41,44] and sigmoid [43]. Several articles also included elastography as a diagnostic technique in other diseases: colitis ulcerosa [38] and tumours (adenocarcinoma, adenoma) [41]. One study by Rustemovic et al. [38] was conducted using a control group. Two studies focused on the paediatric population [36,44]. Other aspects of the analysed papers are presented in Table 2.

### 3.1. Conducted Analysis

All patients included in our analysis were examined utilising transabdominal evaluation of involved segments of the digestive tract. SE was used in 11 studies [31,32,33,34,35,36,37,38,39,41,42], and transrectal SE was described in one study [38]. On the other hand, SWE was utilised in five studies [30,31,40,43,44]. Several articles compared the use of sonoelastography with other diagnostic techniques, namely B-mode ultrasound (US-B) [30,31,32,33,34,35,36,37,39,40,42], CEUS [30,31,33,44], US Doppler [32,35,39] and MRE [34,36,42,44]. In 10 studies, results from imaging diagnostics were verified via histopathology examination [30,31,32,36,38,39,40,41,43].

### 3.2. Aim of the Studies

A study by Lu et al. [30] aimed to demonstrate a correlation between in vivo ileum SWE examination during CEUS (contrast-enhanced ultrasound) and levels of inflammation, fibrosis and muscle hypertrophy in CD. Ding SS et al. [31] assessed the diagnostic potential of strain elastography, acoustic radiation pulses imaging (ARFI) and point transverse wave elastography (p-SWE) in the examination of the most frequent types of bowel strictures in CD. Serra et al. [32] and Baumgart et al. [39] investigated the usefulness of real-time elastography (SE) with the measurement of strain coefficient. They aimed to assess whether it could be used to differentiate inflammatory bowel strictures from fibrosis. Quaia et al. [33] assessed the usefulness of conventional (US-B) and CEUS combined with real-time strain elastography (SE) in distinguishing ileal strictures from fibrosis. Lo Re et al. [34] analysed and compared lesions in the mesenteric and intestine wall in CD using ultrasound, strain elastography and magnetic resonance enterography. Ultrasound imaging of elasticity (UEI) as a method to evaluate the effectiveness of anti-TNF therapy in patients with CD was implemented by Orlando et al. [35]. Fufezan et al. [36] attempted to reveal the role of sonoelastography performed alongside hydrosonography (HS) in the diagnosis of CD activity in children. The authors also suggested a scoring system for disease activity. Fraquelli et al. [37] concentrated on the reliability and repetitiveness of ultrasound imaging of elasticity in the evaluation of ileal fibrosis in CD. Rustemovic et al. [38] evaluated the effectiveness of transrectal SE in differentiating between CD and ulcerative colitis. Chen et al. [40] distinguished between inflammatory and fibrotic lesions in intestinal strictures in patients with CD using real-time ultrasound shear elastography. Havre et al. [41] investigated probes of pathologically constricted intestines in CD using US-B and SE. Sconfienza et al. [42] tested real-time sonoelastography (RTS) in vivo to distinguish fibrosis from inflammatory strictures of the ileum in CD. MRE was used as a reference standard. Goertz et al. [43] assessed the effectiveness of ARFI of the transverse wave velocity of the intestinal wall that was induced in patients with CD. In their study, Thimm et al. [44] compared SWE and CEUS in detecting CD activity.

### 3.3. Activity of the Disease, Operations and Additional Circumstances in Which Studies Were Conducted

Activity of the disease was assessed using the following scales: Crohn’s Disease Activity Index (with an average outcome of 215 points) [31,40], the Harvey-Bradshaw Index (on average 5.47 points) [31,38,40,42,43] and Montreal Classification [34,35,37,38]. In five studies, the activity of the disease was not evaluated [30,33,36,39,44].

Two studies included a routine, one-time imaging assessment using sonoelastography [34,38]. In seven studies [30,31,33,36,42,43,44], the routine examination using this technique was followed by several-week-long patient monitoring as a method of detecting potential complications and necessary operations. In studies in which surgical intervention due to symptomatic bowel stricture was a reference point, sonoelastography was conducted in the perioperative period, which was seven days before surgery on average [32,37,40,44]. Havre et al. [41] performed evaluations of directly resected bowel segments and differentiation between CD-related strictures and tumours. Baumgart et al. [39] conducted pre-, intra- and postoperative assessments. In a study by Orlando et al. [35], sonoelastography was used to assess the effectiveness of anti-TNF therapy and a 52-week follow-up of the patients. Complication-related surgical intervention was present in seven articles, with a mean time of 5.5 months between the sonoelastography and the operation [30,31,33,35,42,43,44].

### 3.4. Experience and Number of Operators

In most studies included in this review, sonoelastography was conducted by only one radiologist [31,33,35,38,40,43]. Data in several studies were collected by more physicians, namely two [32,37,39] to four operators [30]. One study compared performance and accuracy between specialist and resident after five years of training [34]. In a study by Fufezan et al. [36], information about operators was not included. Radiologists’ experience ranged from five years [40] to 35 years [30] according to the selected articles.

### 3.5. Sonoelastography Technique, Region of Interest and Parameters of Sonoelastography

Eleven authors utilised SE techniques [31,32,33,34,35,36,37,38,39,41,42]. Before SE, the majority of authors conducted US-B exploratory evaluation searching for changed bowel segments [31,32,33,34]. Fraqueli et al. [37] and Sconfienza et al. [42] executed US-B and SE including only distal ileum. However, only the former author [37] indicated a specific location in the study (3 cm cephalad from the ileocecal valve). A study by Rustemovic et al. [38] focused exclusively on imaging of the rectum. Fufezan et al. [36] conducted sonoelastography and US-B simultaneously. On the other hand, Baumgart et al. [39] performed US-B followed by SE of changed and unchanged bowel segments for further analysis and comparison. In a study by Orlando et al. [35], SE and USB mode were repeated three times.

Several approaches were implemented to produce adequate SE images. The tissue compression technique was conducted in two studies [31,33]. Conversely, Serra et al. [32] evaluated the deformation of bowel walls induced by vascular pulsations without compression. Other authors utilised a technique of repetitive alternating compressions and decompressions [36,37,39,42]. It was obtained by pressing the head of the US toward the examined area while adjusting the strength and frequency of pressure. The results were displayed as the colour-coded elastogram. Rustemovic et al. [38] utilised a transrectal head in patients without prior bowel preparation. Orlando et al. [35] conducted an evaluation of patients during anti-TNF therapy three times (in weeks 0, 14 and 52). Havre et al. [41] analysed bowel segments resected during surgery. Samples in this study were inserted into a dedicated chamber filled with paraffin wax to reduce the reflection of the ultrasound waves. The tops of the samples were covered with a layer of elastic agar, thus enabling the echoless background.

Colour-coded gradation elastograms after SE analysis were generated in nine articles [31,32,33,34,36,37,39,41,42]. In three of them, elastograms were analysed with the use of a five-colour scale [31,34,37]. Ding et al. [31] categorised elastograms into the following classes: 1—green; 2,3,4—specific colour distribution patterns including blue, green, and red; 5—blue. Classes reflected the degree and distribution of fibrosis. Lo Re et al. [34] labelled colours in the following order: red—healthy tissue; yellow—possible inflammation; green—definitive inflammation; light blue—possible fibrosis; blue—definitive fibrosis. Conversely, Fraquelli et al. [37] proposed a scale constituting five stages, in which the first one is assigned to a red colour representing soft tissues, and stage five is assigned to blue, representing advanced fibrosis.

Three authors assessed only two colours in previously generated elastogram maps, with red representing soft tissues and blue indicating high stiffness tissue [32,33,39]. Fufezan et al. [36] assessed the distortion map with a three-stage colour scale: red—soft tissue; green—intermediate tissue; blue—stiff tissue. Subsequently, the authors classified observed elastograms based on colour patterns into three types. These types were as follows: type A—bowel wall is normal/in remission; B—an inflammatory tissue: thick, irregular with increased vascularisation, but the layer structure remains intact; an acquired image consists of an irregular blue-green pattern; C—thick fibrotic bowel wall, all blue with no visible layer structure. Another study by Havre et al. [42] implemented a sonoelastographic scale introduced by Jansen; previously utilised to evaluate the pancreas [45]. The scale introduces three specific patterns of colour arrangement: type 1—homogenous; type 2—heterogenic; type 3—honeycomb image. Moreover, three-colour groups were determined: A—blue; B—green/yellow; C—red. Diversely, Sconfienza et al. [42] generated a colour-coded elastogram that was converted into a semi-quantitative scale with numerical scores assigned to colours (1—red, representing minimal tissue stiffness; 2—green, representing an intermediate stage of fibrosis; 3—blue, representing maximal stiffness). These scores were multiplied by several assessed sections, eight for each evaluation, resulting in a range from 8 to 24 points.

The strain ratio (SR) was evaluated in seven of the analysed studies [32,35,36,37,38,42]. Both Orlando et al. [35] and Fraqueli et al. [37] calculated SR using surrounding mesenteric tissues as the region of interest (ROI). Moreover, Orlando et al. [35] classified SR = 2 as a cut-off point for severe fibrosis. Serra et al. [32] conducted five loops of elastography for each assessed segment. Each loop consisted of 20 consecutive elastograms and assessed the same strictured bowel section. On each loop, two ROIs were determined. ROI 1 was the reference tissue, and ROI 2 was the upper part of the cross-sectioned gut wall, excluding intestinal contents and surrounding tissues. Fufezan et al. [36] calculated SR using the relation of bowel submucosa to values of anterior abdominal wall muscles. On the other hand, Rustemovic et al. [38] calculated SR comparing measurements of the rectum wall and tissue surrounding the rectum. Baumgart et al. [38] calculated the SR value by obtaining ROI 1 in 2 × 1 cm rectangle of altered bowel segment, in which ROI 2 after excluding intestinal contents and surrounding tissue was determined. In contrast, the methodology used to obtain SR value was not mentioned in the article by Havre et al. [41].

SWE was utilised in five studies [30,31,40,43,44]. All of these authors had conducted conventional US-B prior to SWE measurement to determine bowel segments with potential strictures. Lu et al. [30] examined and measured areas with the thickest bowel wall in each segment or areas with the most notable wall stricture. ROI dimensions were 10 × 5 mm, and it was positioned in axial or longitudinal view with the exclusion of intestinal contents and surrounding tissues. In the axial view, ROI was located between hours 3 and 9 clockwise to seize the largest possible area. Subsequently, ROI was documented as the distance from the ileocecal valve or corresponding fistula for further histological examination. Ding et al. [31] determined the ROI for SWE evaluation in the same area where SE measurement was executed. Moreover, the authors created a smaller ROI within the area with the thickest bowel wall that was subsequently utilised to calculate p-SWE. Chen et al. [40] conducted SWE of constricted intestine segments and compared them with SWE values of non-constricted intestine segments. In this analysis, ROI encompassed the entire bowel wall width. According to both Goertz et. al. [43] and Thimm et al. [44], ROI was the area of the most distinct constriction.

Lu et al. [30] and Ding et al. [31] both utilised ARFI to measure SWE. Furthermore, Lu et al. [30] correspondingly calculated shear wave velocity (ms; SWV). In the study, an assessment was conducted with VTQ and ElastPQ^®^. The quality of the data was evaluated with the use of IQR (interquartile range) divided by median ratio with an assumption that values <0.3 represent correctly performed examination. All results with a ratio of ≥0.03 were excluded from the study. Ding et al. [31] estimated SWVs as an average based on seven ROI measurements. Additionally, in the case of ARFI, the obtained images were assessed based on the distribution of black and white. The authors used a scale ranging from 1 to 5 points (1—white; 5—black).

Chen et al. [40] coded elasticity estimates during SWE to generate quantitative images of SWE, excluding intestinal contents and surrounding tissues. Subsequently, for further statistical analysis, an average from three independent assessments was used. Measurements were not included in the study if the SWE field presented weak or no signals. Similarly, Thimm et al. [44] calculated the average from three measurements, whereas Goertz et al. [43] recorded at least 10 measurements during pauses in patients’ free breathing.

### 3.6. Results Analysis

Inter-operator reproducibility is an important factor for ensuring the method’s usefulness. Dependence on operator expertise and experience was controlled in one study [42]. It demonstrated good agreement (Kappa value = 0.71) in the case of real-time elastography between a more experienced specialist and radiology resident who had been conducting elastography examinations for three years. The lowest Kappa value in the sample was reported by Havre et al. (0.38 for SR elastography) [42]. Other studies uncovered moderate (Quaia et al. [33]) (k = 0.6 for real-time elastography) to excellent agreement (intraclass correlation coefficient = 0.78 for strain ratio SE) [33]. Lo Re et al. [34] stated that disagreement was ‘considered relatively few or minor’. Ding et al. [31] based their study on subjective consensus between two radiologists conducting examinations. Three studies were conducted by only one operator who was blinded [35], non-blinded [43] or without specification [38]. No information about an operator was provided in several studies [30,32,36,40,44].

The diagnostic potential of elastography in detecting fibrosis was assessed by Fraquelli et al. [37]. The authors reported that a measured strain ratio was significantly higher among patients with severe fibrosis (2.4 ± 0.5) than among those with moderate or mild fibrosis (1.5 ± 0.5) and with inflammatory bowel walls (1.2 ± 0.6). Furthermore, SR was revealed to have excellent discriminatory properties (AUROC 0.917 with 95% CI 0.788–1.000).

Several authors evaluated the efficacy of sonoelastography in differentiating between fibrosis and inflammation in bowel wall strictures. Scofienza et al. [42] utilised a semi-quantitative score with real-time sonoelastography, indicating higher values in fibrotic stenosis than in inflammatory stenosis. Two studies demonstrated that sonoelastography can distinguish only fibrotic changes, whereas CEUS [44] or colour Doppler [40] can be applied to detect inflammatory stenosis. Both studies highlight the necessity of combining these methods as complementary diagnostic protocols. Another study by Baumgart et al. [39] suggested significantly lower RTE strain values in bowel segments affected by CD (43.0 ± 25.9) compared to those unaffected by CD (169.0 ± 27.9). Moreover, the authors reported an association between RTE strain values and higher collagen concentration in the tissue, as well as the width of internal muscularis propria and the muscularis mucosae, claiming the feasibility of RTE as a marker of an affected bowel wall in the context of Crohn’s disease. Lu et al. [30] discovered that SWE correlates significantly with peak enhancement in CEUS and E-MRI (DWI), assessing inflammation of the bowel wall. Moreover, authors [30] have reported no correlation between SE and fibrosis. Surprisingly, the significant histopathological correlation that was found only referred to SWE and muscular hypertrophy. One study [33] examined strain elastography as a method to supplement US-B and CEUS. Those methods, when combined, demonstrated higher diagnostic accuracy (but not sensitivity and specificity) than when utilised separately in differentiating fibrotic from inflammatory bowel wall strictures. Finally, Serra et al. [32] claimed that SE cannot discriminate fibrosis nor inflammation in bowel stenosis.

Ding et al. [31] focused on the efficacy of specific types of sonoelastography in differentiating properties of bowel stenosis in CD. This study has emphasised the superiority of p-SWE overstrain elastography and SWE in detecting fibrosis (sensitivity of 95%, specificity of 100%, accuracy of 96%, PPV of 100% and NPV of 95.5%).

In the study by Fufezan et al. [36], the authors attempted to describe bowel wall patterns detected with sonoelastography in CD patients. In contrast to studies aiming to discriminate properties of stenosis, Fufezan assumed a priori that bowel wall patterns can be classified as fibrotic or inflammatory.

Goertz et al. [43] evaluated ARFI as a potential tool for the assessment of inflammation in the bowel wall. In a retrospective part of the study (*n* = 77), ARFI shear-wave values were significantly higher for both ileitis and sigmoiditis when compared to a healthy ileum and colon, respectively. Moreover, ARFI results were positively correlated with bowel thickness and Limberg score. However, in a prospective study, the authors found none of the aforementioned differences. Only one study [36] uncovered a positive correlation between inflammation marker levels (Erythrocyte Sedimentation Rate, CRP and calprotectin) and strain ratio. The authors report that strain ratio is an independent predictor of those markers. In contrast to those findings, studies by Serra et al. [32] and Sconfienza et al. [42] did not reveal any correlations between SE values and inflammation markers.

Sonoelastography was also evaluated as a method of monitoring outcomes of anti-TNF therapy. Orlando et al. [35] uncovered no statistically significant difference in baseline strain ratio and strain ratio after 14 and 52 weeks of therapy. Nevertheless, results of strain ratio were inversely correlated with bowel wall thickness during treatment. Moreover, strain ratio measured at 0 points was lower among patients who achieved transmural improvement, defined as a bowel wall thickness of ≤3 mm. In addition, Serra et al.’s retrospective analysis [32] revealed no significant correlation between previous anti-TNF therapy and strain ratio values.

Two studies confirmed that sonoelastography results may be associated with the probability of future operations. Lu et al. [30] reported that, out of 95 consecutive CD patients that had been included in the study, 15 had a surgical resection, and mean SWE values were higher in this group (2.8 ± 0.7 m/s with a range of 1.5–3.9 m/s vs. 2.2 ± 0.8 m/s with a range of 0.64–4.1 m/s for those who did not have an operation). However, simultaneous and stepwise logistic regression analysis did not prove that SWE is a surgery predicting factor. In the second study, Orlando et al. [35] found that patients with a strain ratio value of ≥2.0 underwent surgery more frequently due to CD complications. However, this study group was limited to patients under anti-TNF treatment.

Fufezan et al. [36] proved that strain ratio is significantly correlated with complications that may necessitate surgery (stenosis, fistula, abscesses). Type B, defined by the authors as an inflammatory wall pattern, was found to be an independent predictor of complications.

Furthermore, sonoelastography was assessed as a tool in the differential diagnosis. Rustemovic et al. [38] demonstrated that the strain ratio measured with transrectal ultrasound elastography is significantly higher among patients with active CD and patients with active ulcerative colitis (median 1.3 vs. 0.49). Moreover, the strain ratio in patients with active CD was higher than in patients in remission (median 1.37; interquartile range [1.2–1.56] vs. median 0.97; IQR [0.54–1.2]) and non-IBD (inflammatory bowel disease) control group. Additionally, CD patients (both with active disease and in remission) exhibited significantly higher strain ratio values compared to non-IBD controls (median 1.18 vs. 0.68).

Another study regarding differential diagnosis by Havre et al. [41] attempted to discriminate CD-related strictures from malignant and benign tumours (adenocarcinoma and adenoma). The authors reported no significant differences between these lesions in any of the SR elastography parameters, although both CD strictures and tumours displayed higher density than surrounding tissues.

## 4. Discussion

Elastography-based imaging techniques have received substantial attention in recent years for the non-invasive assessment of tissue mechanical properties. While ultrasound elastography has yielded promising results for the assessment of liver fibrosis, new applications in breast, thyroid, prostate, kidney and lymph node imaging are emerging [46,47]. Liver elastography can be useful both in the diagnosis of liver fibrosis and in monitoring treatment, as in one study by Facciorusso et al. [48] In their study, they confirmed the relationship between antiviral therapy in patients with hepatitis B and liver stiffness and proved that antiviral therapy is associated with a progressive decrease in liver stiffness, particularly among patients with hepatitis without high baseline levels of alanine aminotransferase and viremia. One of the newest applications of elastography is intestinal elastography.

In the case of the incorporation of new methods into clinical use, the further assessment and comparison in relation to conventional methods is necessary. For clinicians, the most important question concerns the status of sonoelastography among other non-invasive diagnostic methods for Crohn’s disease. It has been predicted that sonoelastography may be able to replace CT and MR among patients with CD. Patients with Crohn’s disease, along with the disease progression, are exposed to an increasing number of diagnostic tests assessing the effectiveness of treatment or complications. It is therefore important to ensure that these patients receive adequate radioprotection and to avoid the use of ionising radiation examinations such as CT. In addition to the high dosage of ionising radiation, CT enterography also has difficulties in detecting fibrotic stenosis [49].

MRE, which is free of ionising radiation, may be the most accurate and most broadly applicable available approach for stricture differentiation. Although MRI enterography indicates high sensitivity and accuracy in detecting bowel strictures, similarly to CT, it has problems distinguishing between inflammatory and fibrotic strictures [50]. Moreover, MRI remains a time-consuming, relatively expensive examination with limited availability in various centres and limited patient comfort. For all these reasons, transabdominal US has emerged as an increasingly important imaging tool which is easily available, fast, inexpensive and repeatable, while maintaining the patient’s comfort during the examination. In turn, Reiter et al. [51] demonstrated the feasibility of MR elastography of the gut and showed excellent diagnostic performance in predicting IBD. In their study, Lo Re et al. [34] compared the feasibility of sonoelastography and MR in differentiating the type of strictures in CD, with a significant correlation between the results of these two methods. However, these findings are not conclusive regarding the possible replacement of MR by sonoelastography as the only diagnostic method. Nevertheless, SE may be utilised as a complementary technique, specifically in the assessment of inflammatory and fibrotic lesions in the mesentery. A study by Mazza et al. [52] revealed substantial agreement between MRE and SE in the assessment of fibrotic changes in the intestines over the course of CD; it also suggested their potential predictive role in the prediction of surgery or hospitalisation. The authors suggest that both techniques may be adopted for general use in the future. Due to the comparable results of both methods, they also suggest that when selecting a given test technique, consider using lower costs and saving time with the use of SE.

Similar conclusions have been drawn from studies comparing CEUS and sonoelastography. SWE allows researchers to quantitatively assess the stiffness of the intestinal wall. It is an appropriate diagnostic choice to detect and evaluate strictures associated with fibrosis and hypertrophy of smooth muscles in the bowel wall. CEUS is limited to the qualitative assessment of inflammation. Furthermore, it was indicated that higher values of SWE correlate significantly with bowel wall stiffness and suggest an inverse relationship with CEUS results [30,44].

In clinical practice, CEUS and sonoelastography may be regarded as complementary techniques that result in a more accurate assessment and classification of lesions. This is especially promising in the case of overlapping inflammation and fibrosis. It has been proven that SE has lower sensitivity, but higher specificity in comparison to CEUS in detecting intestinal fibrosis. However, each method separately exhibited minimal accuracy. The combination of sonoelastography and CEUS resulted in low total sensitivity and specificity, but an increase in accuracy was observed [33].

Furthermore, sonoelastography may be useful in assessing the aetiology of strictures. The inflammatory or fibrotic origin of it can be detected, owing to the difference in tissue stiffness, which is low in inflammation and high in fibrosis [53,54]. Moreover, patients with active CD have higher reflection factors in affected regions compared to unchanged ones relative to patients in remission [38].

Although it has been proven that sonoelastography correlates with endoscopy findings in the diagnosis of ulcerative colitis, our analysis presented contradictory findings in differentiating stricture aetiology in CD [55]. The majority of the included studies indicate that it is possible to successfully detect the fibrotic origin of strictures, but not inflammatory ones. Additionally, one study by Serra et al. [32] revealed a lack of diagnostic efficacy of elastography in detecting and classifying strictures in CD.

From a clinical perspective, a significant advantage of elastography is the immediate imaging of present lesions. However, reproducibility and appropriate interpretation remain questionable, with the strong necessity of establishing adequate and standardised classification criteria. In our analysis, these criteria differ significantly between researchers. Moreover, further studies do not confirm their efficacy and do not validate these criteria in light of the proper standardisation process. This problem refers to both colour-coded elastograms and strain elastography. In the former case, there are differences in the evaluated colour patterns. In the case of SE, researchers compared ROIs with mesenteric fat [35,37], unaffected intestine or abdominal muscles [36], which resulted in differences in the SR values obtained in several studies. The selection of the appropriate tissue for comparison is also a limiting factor which influences the method itself. Mesenteric fat surrounding the inflamed section of the intestine may be affected by the disease itself, rendering it impossible to calculate an appropriate SR. In the abdominal muscles, age-related fat changes may occur, and muscle structure may vary between patients depending on activity, disease severity, nutritional status and sex. On the other hand, selecting the appropriate, healthy section of the intestine becomes problematic when a large section of the intestine is affected by disease due to peristaltic movements. To overcome this problem, a non-dependent on surrounding or affected-to-healthy ratio tissues method could be implemented; for example, this could be similar to that introduced by Hitachi and based on RTE, liver fibrosis index (LFI). This semi-quantitative method was implemented to evaluate the liver fibrosis without the impact of inflammatory changes; to compose an analogical tool with the use of multiple regression, a large sample of SE images and reference method results would need to be obtained [56]. For authors who have used colour-coded elastograms, there is a large discrepancy in the classification of different tissue types. Some authors used a five-colour scale [31,34,37], others a three-colour scale [36,41,42], and still others a two-colour scale [32,33,39]. In addition, different authors using the same number of colours classify completely differently what a given colour corresponds to and use drastically different interpretations of colour maps. Therefore, there is an urgent need to create a unified and validated assessment scale that can be used on various types of ultrasound devices and in various centres around the world. Thus far, however, no study has been developed comparing the effectiveness of different classification systems using SE, and the need for such analysis is increasing. From the clinician’s perspective, the most easily interpreted and practicable system at present is the two-colour system, in which blue represents fibrosis or interstitial oedema, and red represents inflammation. Despite the disadvantages mentioned earlier, we believe that SE, with visual observation of elastography colour patterns representing the bowel wall, is easily performed and provides relevant information.

Given the above limitations, some authors have attempted to standardise the systems for assessing intestinal elastograms. In their study, Marin et al. [57] proposed creating a scale evaluating various ultrasound parameters: bowel wall thickness score (BWT score), Limberg score, ARFI score and disease extension score. The composite score above had effective results correlating with IBD severity scales (Harvey-Bradshaw Index and Mayo score) and inflammatory markers. For CD, a cutoff value of eight points can identify the active disease with a sensitivity of 81.81% and a specificity of 83%.

Compared to SE, SWE is a reproducible, objective and quantitative technique for measuring organ stiffness. It has been argued that in the case of SWE measured with ARFI, the bowel wall is too thin, while the measurement window is too wide. Thus, it also contains surrounding tissues and intestinal contents. This may cause measurement errors. Authors utilising SWE have adopted many solutions to address this problem including several methods of variables measurement [58]. This problem could be addressed via the usage of systems that offer the ROI size change option (such as Hologic, Philips or Samsung), but it would result in new problems with standardisation, as SWE measurements are both vendor- [59] and ROI size-dependent [58,60]. Without further studies comparing different measurement methodologies to identify one with the highest accuracy and reproductivity, it is difficult to recommend the best approach. Furthermore, the heterogeneity of the study protocols and applied methods result in the inability to conduct a reliable meta-analysis. For this reason alone, performing subsequent research using the same methodology appears to be justified.

The main limitation of elastography, both SWE and SE, is interpretation subjectivity due to the diagnostic method itself. Similarly, as in US, the quality of obtained images is operator dependent. Moreover, subjective evaluation influences the reproducibility of the technique. However, the degree of this problem is beyond current analysis due to inter-operator agreeability in the studies that changed from weak to excellent. Furthermore, several authors did not address this important aspect. The quality of images is also patient dependent. The outcome of the examination relies on proper preparation and the presence of gas in the intestinal lumen. Some authors have suggested that large blood vessels close to ROI diminish the accuracy of elastography. Furthermore, constant intestinal movements are an important diagnostic impediment. To evade increased mobility and artefacts as a result of it, radiologists may have to introduce spasmolytic premedication. However, it is important to note that movements of fibrotic bowel segments are significantly decreased and easier to evaluate in SE [61]. A noteworthy method of bowel movements measurement is strain rate imaging (SRI), which visualises and computes peristaltic activity and its medication response [62]. Perhaps a combination of the above could offer interesting results.

Another limitation of the analysed studies is a specific group of CD patients taken into consideration. In several studies, patients were assessed prior to the surgical operation, especially in studies that correlated elastography results with histopathological assessment. This poses a question regarding whether the results of these studies can be easily generalised to other patients with a milder course of the disease without indications for surgical intervention.

In addition, elastography does not allow for differentiation with other intestinal pathologies or for a histological diagnosis, but only information about the stiffness of the intestinal walls [63]. Havre et al. [41] demonstrated that the SE and SR measurements and visual assessment did not distinguish the strictured Crohn’s lesions from adenocarcinomas in excised intestinal specimens. It was found that a small number of adenomas are much softer than adenocarcinomas, and the severity or degree of the tumour did not significantly affect the results of elastography. Given the oncological risk of CD, there is a chance of confusing fibrotic or inflammatory stenosis with neoplastic stenosis. However, this study was conducted using a small number of patients (*n* = 27) and lesions (18 adenocarcinomas, 4 adenomas); thus, the results require confirmation by other researchers using larger groups of patients.

To our best knowledge, there are only two studies that have focused on the use of SE in paediatric patients with a diagnosed CD. This includes one study that was a series of three case studies. Thus, the feasibility of elastography in this group of patients has not yet been thoroughly analysed. However, both of the aforementioned studies report the efficacy of sonoelastography in paediatric patients.

## 5. Conclusions

Elastography is an easy, reproducible and non-invasive method that has yielded promising results in assessing the severity of Crohn’s disease. Despite small sample sizes, in the studies conducted thus far, it has found its place in the recommendations of The European Federation of Societies for Ultrasound in Medicine and Biology Guidelines and Recommendations for the Clinical Practice of Elastography in Non-Hepatic Applications, which indicate intestinal elastography as the only method capable of differentiating lesions inflammatory and fibrotic diseases in CD. Among other diagnostic methods used in CD, elastography appears to be insufficient to be used independently, but it seems valuable as a supplementary method. Additionally, due to its ease of use and high availability, it can be highly useful in monitoring previously detected changes. Despite the advantages, it should be remembered that both SE and SWE do not distinguish neoplastic stenosis. Additionally, to facilitate the use of SE and SWE, standardised measurement strategies and appropriate scales should be created for uniform results. There is an increasing need for a cross-platform standardisation that would allow comparable results to be obtained in various centres with different US devices. At this point, the knowledge of elastography in the paediatric population is insufficient, but the preliminary results are promising.

## Figures and Tables

**Table 1:** Summary of the articles included in the analysis.

Authors	Year	Type	Number of Participants	Population	Age (Years), Mean (SD)	Duration of the Disease (Years), Mean (SD)	Aim of the Research	Elastography Technique	Additional Methods	Histopathological Assessment	Device	Operator’s Experience (Years)	Number of Operators
Cathy Lu et al. [24]	2017	Prospective	95 (80 no surgery; 15 surgery)	Adults	Surgery: 41 ± 14.4; no surgery 46.1 ± 13.7	12.7 ± 13.9	To correlate SWE of ileal Crohn’s disease in vivo to CEUS peak enhancement and pathology grades of inflammation, fibrosis and muscular hypertrophy.	SWE	CEUS, US B-Mode	Yes	Acuson S3000 (Siemens Medical Solutions USA, Inc) or Philips Epiq 5 (Philips Healthcare)	35 (all together)	4
Ding SS et al. [25]	2019	Prospective	25 (21 inflammatory; 4 fibrotic)	Adults	Inflammatory strictures: 40 ± 14; fibrotic strictures: 35 ± 19	Inflammatory strictures: 7 ± 6.7; fibrotic strictures: 7.8 ± 5.7	To evaluate the diagnostic performance of strain elastography, acoustic radiation force impulse (ARFI) imaging and point shear wave elastography (p-SWE) to assess the predominant types of intestinal stenosis in Crohn’s disease.	SE, SWE ARFI, p-SWE	US B-Mode	Yes	S2000 ultrasound scanner (Siemens Medical Solutions, Mountain View, CA)	10 (ultrasonography), 3 (sonoelastography)	1
Serra C et al. [26]	2017	Prospective	26	Adults	35.5 ± 11.0	11.7 ± 7.5	To measure bowel wall stiffness in stricturing Crohn’s patients using in vivo RTE and to evaluate its role in distinguishing the extent of fibrosis and inflammation assessed by histology. In addition, the relationship between US, colour-Doppler and CEUS, and the histological features of the stenotic bowel wall was assessed.	RT-SE	US B-Mode, CEUS	Yes	iU22 Philips (Philips, Bothell, WA, USA); Philips 5–12 MHz linear transducer	Strong experience	2
Quaia E et al. [27]	2018	Prospective	20	Adults	40.2 ± 10.22	6	To prospectively assess the feasibility of conventional B-mode ultrasound (US) and CEUS combined with real-time SE in the differentiation of inflammatory from fibrotic ileal strictures among patients with CD based on visual analysis.	RT-SE	US B-Mode, CEUS	No	iU22 xMATRIX Ultrasound System (Philips Healthcare, Bothell, WA, USA); broadband 256-element linear-array transducer (L12-5, 5–12 MHz, 50 × 10 mm^2^)	10	1
Lo Re G et al. [28]	2017	Prospective	35	Adults	33.12	No data	To assess whether SE and DWI could be used to detect mesenteric and bowel wall fibrosis and differentiate it from oedematous/inflammatory changes.	SE	MRE, US B-Mode	No	Samsung RS80A (Samsung Medison Co. Ltd.); linear-array transducer (EUP-L74M, 5–13 MH Samsung)	15; 5	2
Orlando S et al. [29]	2018	Prospective	30	Adults	38.8 ± 14.5	9.8 ± 7.7	To investigate whether the severity of intestinal fibrosis, as evaluated by UEI, would predict the therapeutic outcomes of CD patients undergoing treatment with anti-TNF antibodies. The relationship between intestinal fibrosis and anti-TNF-induced transmural healing was also assessed as a secondary outcome.	SE	US B-Mode, Power Doppler	No	Philips iU22 apparatus (Philips Ultrasound; Philips Healthcare, Bothell, WA); multi-frequency convex [C5-2, 5–2 MHz] and a linear array transducer (L12-5, 12–5 MHz)	Strong experience	1
Fufezan O et al. [30]	2015	Prospective	14	Paediatric	12 ± 3.67	-	The aim of this study is to determine whether SE of the bowel wall, in addition to hydrosonography (HS) of the colon, is a useful tool for assessing and monitoring paediatric patients with CD and to propose an SE scoring system for the assessment of CD activity.	SE	MRE, US B-Mode	Yes	Toshiba Xario V 2.0 ultrasound machine with a linear probe 14–7 MHz	No data	No data
Fraqueli M et al. [31]	2015	Prospective	43 (23 surgery; 20 inflammatory)	Adults	Surgery: 40 ± 12; inflammatory 36 ± 13	Surgery 8 ± 8; inflammatory 8 ± 7	To assess the correlation between UEI results and bowel wall fibrosis at histology, to verify the feasibility and reproducibility of the technique, and to identify the main determinants of UEI results in patients with ileal CD. The performance of standard bowel US parameters in diagnosing severe ileal fibrosis was assessed as a secondary objective.	SE	US B-Mode	Yes	Philips iU22 apparatus (Philips Ultrasound; Philips Healthcare, Bothell, WA); multifrequency convex (C5-2, 5–2 MHz) and a linear array (L12-5, 12–5 MHz) transducer	No data	2
Rustemovic N et al. [32]	2011	Prospective	30	Adults	30.64	5.5	To assess the role of transrectal ultrasound elastography to distinguish between CD and UC.	Transrectal SE	None	No	Linear echo-endoscope (Pentax FG-38 UX); probes of 7, 5–12 MHz (Hitachi EUB 8500)	No data	1
Baumgart DC et al. [33]	2015	Prospective	10	Adults	49	11.6	The consecutive cohort included consenting adult patients with established Crohn’s disease and symptomatic stenosis that required surgery on the basis of current guidelines and was confirmed by both a gastroenterologist and a surgeon.	RT-SE	US B-Mode, Doppler	Yes	Linear-array transducer (EUP-L74M, 5–13 MHz, 50 × 10 mm^2^; Hitachi)	15; 20	2
Chen YJ et al. [34]	2018	Prospective	35	Adults	34.8 ± 11.3	2.7 ± 2.9	To determine whether shear-wave elastography (SWE), a novel modification of elastography, quantifying tissue stiffness, could differentiate between inflammatory and fibrotic components in strictures of patients with CD.	SWE	US B-Mode	Yes	Aixplorer US system (SuperSonic Imagine S.A., Aix-en-Provence, France); convex broadband probe (SC6-1) and linear array probe (SL 15-4)	5	1
Havre RF et al. [35]	2014	Prospective	27	Adults	No data	No data	To evaluate whether RTE could distinguish between lesions caused by inflammation and malignant neoplastic lesions using qualitative and semi-quantitative methods for strain assessment. Furthermore, interactions between strain ratio (SR) and changes in the elasticity dynamic range (E-dyn) was assessed. Finally, a correlation analysis of elastography results, a histological semi-quantification of fibrosis, inflammation parameters and tumour description were conducted.	RT-SE	US B-Mode	Yes	Hitachi Hi Vision 900 ultrasound scanner with software version V16–04 STEP 2; L54 M linear probe with frequencies 9–13 MHz (Hitachi Medical Corporation, Tokyo, Japan)	No data	1
Sconfieza LM et al. [36]	2015	Prospective	16	Adults	41	10.8	To ascertain whether RTS could differentiate fibrotic from inflammatory strictures in vivo in patients affected by terminal ileum CD, using MRE as a reference standard.	RTS	US B-Mode, MRE	No	High-resolution linear broadband array transducer (13–6 MHz on MyLab 70 XvG system, Esaote, Genova, Italy)	10	1
Goertz RS et al. [37]	2018	Prospective and retrospective group	98 (77 retrospective group; 21 prospective group)	Adults	Retrospective: 37; prospective: 41	No data	To evaluate ARFI shear-wave velocities in patients with CD. ARFI measurements of the stomach, the terminal ileum, and the sigmoid were compared and correlated with ultrasonic B-mode findings of bowel wall inflammation and with CD clinical disease activity.	SWE ARFI	US B-Mode	Yes	Acuson S2000 (Siemens Medical Solution, software version VB21A, Erlangen, Germany); the 9 MHz linear transducer	6	1
Thimm MA et al. [38]	2019	Series of case studies	3	Paediatric	17.67	No data	To evaluate disease activity in patients with CD including acute inflammation, chronic inflammation with stricture formation, and a post-surgical fibrotic stricture. Moreover, an interpretation of CEUS kinetic parameters and elastography values in the evaluation of CD activity was performed.	SWE	CEUS, US B-Mode, MRE	No	EPIQ scanner (Philips Healthcare, Bothell, WA, USA); a broadband 162-element curved array transducer (C5-1, 1–5 MHz, and 55.5 mm)	20	1

Abbreviations: ARFI—Acoustic Radiation Force Impulse; CEUS—contrast-enhanced ultrasound; CD—Crohn’s disease; DWI—diffusion-weighted imaging; p-SWE—point shear wave elastography; RTE—real time elastography; RTS—axial-strain real-time sonoelastography; RT-SE—real time strain elastography; SE—strain elastography; SWE—shear wave elastography; UC—ulcerative colitis; UEI—ultrasound elasticity imaging.

**Table 2:** Diagnostic feasibility in differentiating inflammation and fibrosis.

Authors	Inflammatory Changes	Fibrotic Changes	Stricture/Bowel Wall	Type of Sonoelastography
Cathy Lu et al. [24]	+/−	0	Stricture	SWE
Ding SS et al. [25]	+	+	Stricture	SE, SWE ARFI, p-SWE
Serra C et al. [26]	−	−	Stricture	RT-SE
Quaia E et al. [27]	+	+	Bowel wall	RT-SE
Lo Re G et al. [28]	+	+	Bowel wall	SE
Orlando S et al. [29]	+	−	Stricture/bowel wall	SE
Fufezan O et al. [30]	+	+	Bowel wall	SE
Fraqueli M et al. [31]	+	+	Bowel wall	SE
Rustemovic N et al. [32]	+	0	Bowel wall	Transrectal SE
Baumgart DC et al. [33]	0	+	Stricture	RT-SE
Chen YJ et al. [34]	−	+	Stricture	SWE
Havre RF et al. [35]	−	+	Stricture	RT-SE
Sconfieza LM et al. [36]	+	+	Stricture	RTS
Goertz RS et al. [37]	+/−	0	Bowel wall	SWE ARFI
Thimm MA et al. [38]	−	+	Stricture	SWE

Legend: + feasible, − not feasible, 0 no data. Abbreviations: SWE—shear wave elastography; SE—strain elastography; ARFI—Acoustic Radiation Force Impulse; p-SWE—point shear wave elastography; RT-SE—real time strain elastography; RTS—axial-strain real-time sonoelastography.

## Data Availability

Search results are available from the authors.

## Supplementary Materials

The following are available online at https://www.mdpi.com/article/10.3390/diagnostics11091609/s1, File S1: PRISMA 2020 Checklist, File S2: Queries to the searched databases with filters.
