# Supplementary material for: Shear Wave and Strain Elastography in Crohn’s Disease—A Systematic Review
Source: Diagnostics (Basel). 2021 Sep 3;11(9):1609. doi: 10.3390/diagnostics11091609 (PMC8468946; doi:10.3390/diagnostics11091609)
Supplement: Supplementary file 1 [file diagnostics-11-01609-s001.zip › Supplement 2.pdf]

Supplement 2. Queries to the searched databases with filters.

PubMed

Ultrasound Shear Wave OR elastography OR elastograms OR elastographies AND Crohn disease

Filters: abstract, only human, <10 years

((("diagnostic imaging"[MeSH Subheading] OR ("diagnostic"[All Fields] AND "imaging"[All Fields]) OR "diagnostic imaging"[All Fields] OR "ultrasound"[All Fields] OR "ultrasonography"[MeSH Terms] OR "ultrasonography"[All Fields] OR "ultrasonics"[MeSH Terms] OR "ultrasonics"[All Fields] OR "ultrasounds"[All Fields] OR "ultrasound s"[All Fields]) AND ("shear"[All Fields] OR "sheared"[All Fields] OR "shearing"[All Fields] OR "shearings"[All Fields] OR "shears"[All Fields]) AND "Wave"[All Fields]) OR ("elasticity imaging techniques"[MeSH Terms] OR ("elasticity"[All Fields] AND "imaging"[All Fields] AND "techniques"[All Fields]) OR "elasticity imaging techniques"[All Fields] OR "elastographies"[All Fields] OR "elastography"[All Fields]) OR ("elasticity imaging techniques"[MeSH Terms] OR ("elasticity"[All Fields] AND "imaging"[All Fields] AND "techniques"[All Fields]) OR "elasticity imaging techniques"[All Fields] OR "elastogram"[All Fields] OR "elastograms"[All Fields]) OR ("elasticity imaging techniques"[MeSH Terms] OR ("elasticity"[All Fields] AND "imaging"[All Fields] AND "techniques"[All Fields]) OR "elasticity imaging techniques"[All Fields] OR "elastographies"[All Fields] OR "elastography"[All Fields])) AND ("crohn disease"[MeSH Terms] OR ("crohn"[All Fields] AND "disease"[All Fields]) OR "crohn disease"[All Fields])

Embase

Filters: abstract, only human, <10 yers

('ultrasound shear wave' OR (('ultrasound'/exp OR ultrasound) AND shear AND ('wave'/exp OR wave)) OR 'elastography'/exp OR elastography OR elastograms OR elastographies) AND ('crohn disease'/exp OR 'crohn disease')

Scopus

Filters: abstract, only human, <10 yers, articles, review

((TOPIC: (((Ultrasound Shear Wave OR elastography) OR elastograms) OR (elastographics AND crohn disease)) AND LANGUAGE: (English)) AND DOCUMENT TYPES: (Article))

TITLE-ABS-KEY ((ultrasound AND shear AND wave OR plastography OR histograms OR cartographic) AND crohn AND disease)
